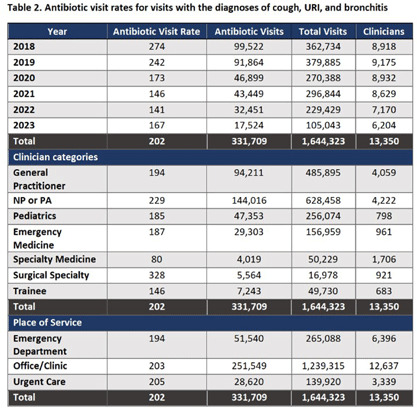# Use of Statewide All-Payers’ Claims Data to Create Outpatient Antibiotic Use Dashboards: A Public Health Stewardship Initiative

**DOI:** 10.1017/ash.2025.246

**Published:** 2025-09-24

**Authors:** Kelly Biermann, Beth Ellinger, Dana Richardson, Mariah Welke, Lindsay Taylor

**Affiliations:** 1 University of Wisconsin; 2 Wisconsin Department of Health Services; 3 The Wisconsin Health Information Organization

## Abstract

**Background:** The Centers for Disease Control and Prevention (CDC) Core Elements of Antibiotic Stewardship for Health Departments includes tracking and reporting of antibiotic use (AU). To support outpatient AU tracking and reporting, the Wisconsin Department of Health Services (DHS) leveraged all-payers’ claims data to create health care organization-specific outpatient AU dashboards with benchmarked measures. **Methods:** DHS contracted with the Wisconsin Health Information Organization’s to access their database to review all payers’ claims data from 2018–2023, which included medical encounter and pharmaceutical claims. Visits were included if they occurred at a clinic (in-person or virtual), urgent care, or emergency department in Wisconsin. Antibiotic visits were defined as an outpatient visit associated with a filled oral, systemic antibiotic prescription ordered up to three days after the encounter. Antibiotic visits were normalized by all outpatient visits as a rate per 1,000 visits. A quality measure of antibiotic visits for respiratory tract infection (RTI) was developed using the CDC’s tier 3 ICD-10 codes representing cough, upper RTI, or bronchitis without co-occurring ICD-10 code for other infection. Antibiotic visit rates were then summarized at the health care organization level, with additional stratification by place of service, diagnosis, patient age, and provider type. **Results:** From 2018–2023, there were over 59 million outpatient visits in Wisconsin by over 20,000 different clinicians from 57 organizations. Statewide benchmarks were developed for all diagnoses and RTI antibiotic visit rates (Tables 1 and 2). Visits by surgeons and advanced practitioners had the highest rates of antibiotic prescribing for tier 3 RTIs; rates were similar across places of service. Rates per individual prescriber were visually compared to the statewide and organization benchmarks within dashboards (Figure 1). **Conclusion:** All-payers’ claims data provides a statewide data source to develop antibiotic visit rates, including a quality measure for visits associated with RTI. Overlying statewide and organizational medians provides a benchmark to identify outliers for further stewardship interventions. Piloting these dashboards will help improve access to AU data.

**Figure:**
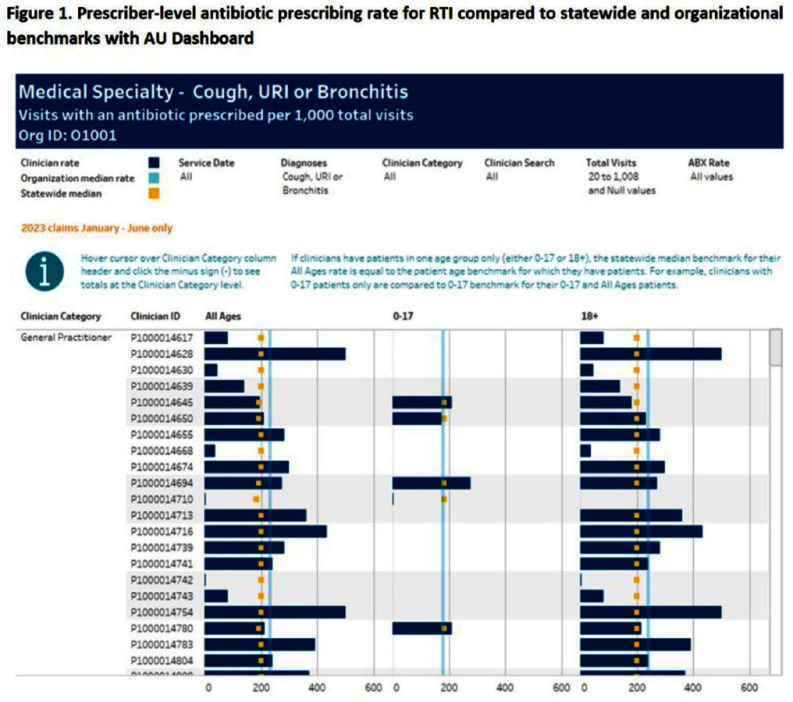

**Figure:**
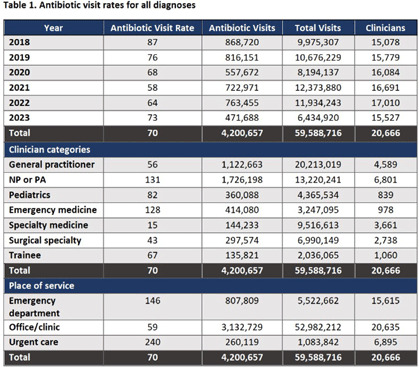

**Figure:**